# No one accelerometer-based physical activity data collection protocol can fit all research questions

**DOI:** 10.1186/s12874-020-01026-7

**Published:** 2020-06-03

**Authors:** Patrick Bergman, Maria Hagströmer

**Affiliations:** 1 Department of medicine and optometry, eHealth Institute, Linnaeus University, 39182 Kalmar, Sweden; 2 Karolinska Institutet, Department of Neurobiology, Care Sciences and Society, Division of Physiotherapy, Alfred Nobels Allé 23, 141 83 Huddinge, Sweden; 3 Karolinska University Hospital, Allied Health Professional Function. Medical unit Occupational Therapy and Physiotherapy, 17176 Stockholm, Sweden; 4 Department of Health Promotion Sciences, Sophiahemmet University, 114 86 Stockholm, Sweden

## Abstract

**Background:**

Measuring physical activity and sedentary behavior accurately remains a challenge. When describing the uncertainty of mean values or when making group comparisons, minimising Standard Error of the Mean (SEM) is important. The sample size and the number of repeated observations within each subject influence the size of the SEM. In this study we have investigated how different combinations of sample sizes and repeated observations influence the magnitude of the SEM.

**Methods:**

A convenience sample were asked to wear an accelerometer for 28 consecutive days. Based on the within and between subject variances the SEM for the different combinations of sample sizes and number of monitored days was calculated.

**Results:**

Fifty subjects (67% women, mean ± SD age 41 ± 19 years) were included. The analyses showed, independent of which intensity level of physical activity or how measurement protocol was designed, that the largest reductions in SEM was seen as the sample size were increased. The same magnitude in reductions to SEM was not seen for increasing the number of repeated measurement days within each subject.

**Conclusion:**

The most effective way of reducing the SEM is to have a large sample size rather than a long observation period within each individual. Even though the importance of reducing the SEM to increase the power of detecting differences between groups is well-known it is seldom considered when developing appropriate protocols for accelerometer based research. Therefore the results presented herein serves to highlight this fact and have the potential to stimulate debate and challenge current best practice recommendations of accelerometer based physical activity research.

## Background

Measuring physical activity and sedentary behavior accurately remains a challenge. An important development in this field has been the widespread use of physical activity monitors such as accelerometers to objectively quantify the level and pattern of physical activity and simultaneously also sedentary behavior. When using accelerometry in research, a protocol is required that strictly determines for example number of days and how the accelerometer will be worn, what cut-offs for physical activity levels will be used and how the physical activity will be summarized [1–4].

**Fig. 1:**
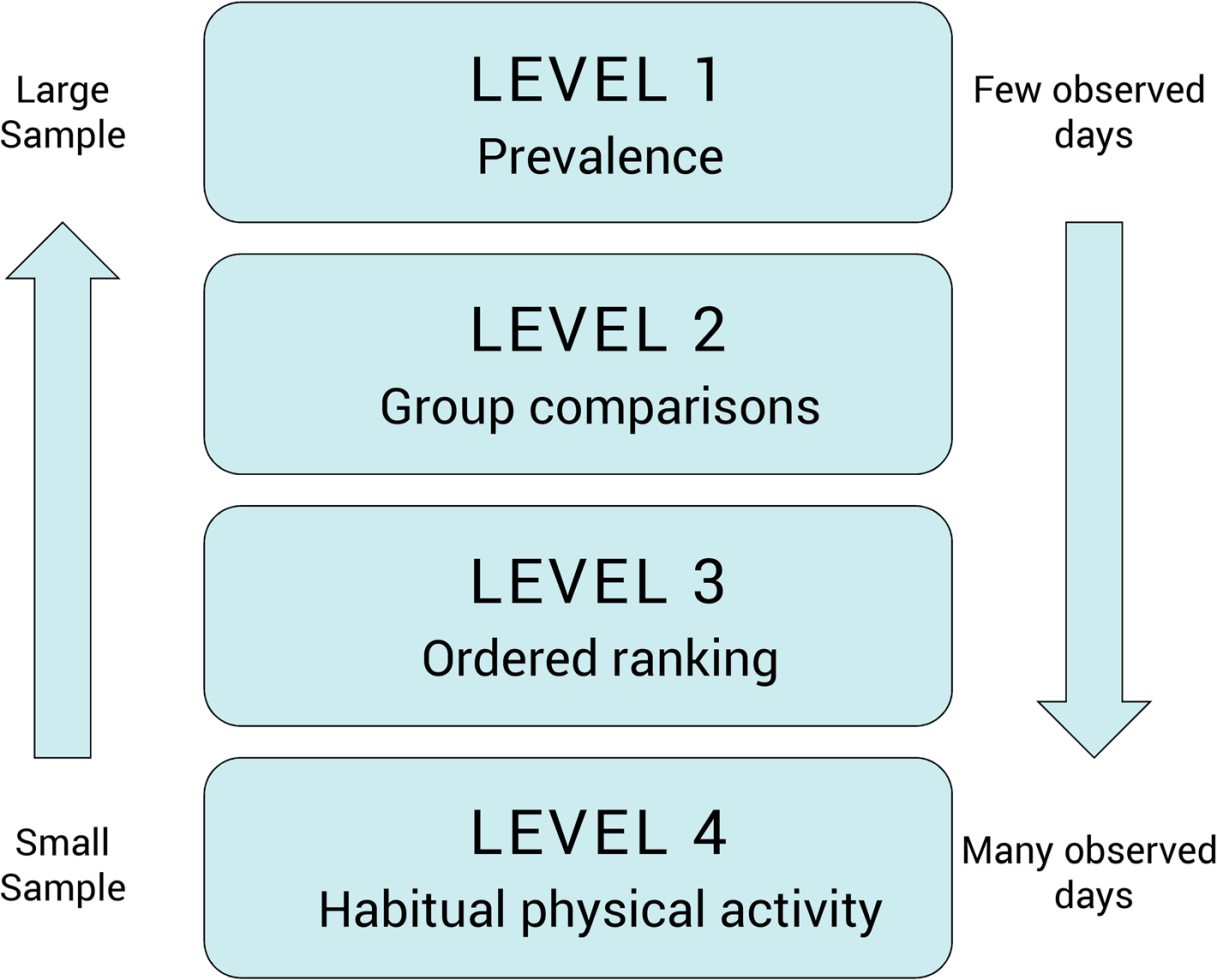
The figure illustrates the different levels according to their research questions and with the association between sample size and the number of observed days in each subject

One of the most important factors included in such a protocol is the number of days that a subject should be monitored as this cannot be changed afterwards. This is central since the number of repeated observations will influence on the measurement error and in extension on the outcome. Several studies have examined this either by using the “Spearman-Brown approach” (e.g. [5–15], or by using the “Generazibility theory approach (G-theory)” (e.g. [16–19]). These studies show relatively consistently that 3–5 days of repeated observations is sufficient to achieve reliability coefficients of around 0.7, and have served as a basis for current guides on best practices in accelerometer based physical activity research which recommend a 7-day measurement period to have some margin to compensate for days during which the accelerometer was not worn [1–4]. However, one thing that is often neglected is that the outcome of the previously mentioned studies has to be conditioned on “*how many days are needed to do what and to what precision*” [20]? The appropriate number of days depends on the aim of the study. Is the aim to estimate physical activity on group level or on individual level? These two levels require different numbers of monitored days. In nutritional epidemiology, which shares many of the measurement problems with physical activity research, four levels of measurement (Fig. 1) relating to different types of research questions have been described [21].

The first level is when a researcher is interested to determine the mean level of physical activity in a group, such as the case when estimating the prevalence of physical activity in large populations or when following trends over time. The second level concerns questions when the mean and distribution of physical activity in a group, for example when evaluating a randomised controlled experiment by comparing treatment groups, is of interest. Level three is when the ranking of individuals according to their level of physical activity is of interest. This level is relevant to epidemiological research in which the aim often is to create groups that are heterogeneous with regards to different levels of physical activity (e.g. tertiles of physical activity), but where the absolute value may not be as important. The fourth level is when a researcher is interested to determine the habitual (usual) mean level of physical activity in an individual, for example when giving individualized advice or to perform analysis correlating a biomarker measured on individual level with the activity level of the same individual. Here, it is usually important that the absolute value of physical activity is measured accurately.

As previously described several studies have already investigated some of these issues. The previous published studies, independently if the “Spearman-Brown” or “G-theory” approach have been used, provide information about the number of days needed to a given reliability answer questions at the third level and to the best of our knowledge, a previously published study from us remains the only that has investigated the reliability needed for questions relating to level four [22], while no studies could be found that have dealt with research questions at level one or two. Thus, there is a gap in knowledge regarding how to best plan a study aiming at answer research questions at the first two levels.

For the first two levels minimising the standard error of the mean (SEM) is important. The SEM is an estimate of how far the sample mean is likely to be from the population mean and is used for example when calculating confidence intervals around the mean. SEM is also important when calculating if one group is significantly different from another group when using for example a Students t-test. It is generally accepted that there are two ways to reduce the SEM; either by increasing the sample size, or by increasing the number of observations within each subject. Therefore, the aim of this study is to investigate how different combinations of sample sizes and the number of monitored days influence on the magnitude of the SEM in accelerometer based physical activity research.

## Methods

### Sample and study design

The study used a convenience sample consisting of university students and staff as well as staff recruited from nearby work-sites. The participants were approached by e-mail. They were sent information regarding the nature of the study and what was expected of them. If they were interested in participating in the study they were asked to reply to the e-mail. A total of 61 subjects agreed to participate and were given accelerometers.

To capture more of the natural variation in physical activity, such as the week-to-week variation [5], than is typically done, a four-week long data collection period instead of the standard seven-day one was used. The participants were asked to wear the accelerometer during waking hours, only taking it off during water-based activities and while sleeping. The participants received a total of three visits from a member of the research group. At the first visit the participants got a brief explanation of how the accelerometer works and were instructed on how to properly position the accelerometer at the right hip. They were also instructed that all information collected during the study would be kept confidential and that they could leave the study at any time without having to provide a reason. The second visit took place approximately 2 weeks after the first visit, during which the batteries of the accelerometer were charged. This took approximately 2 h. The third visit took place after an additional 2 weeks and at this time point the accelerometer was returned and the participants were asked to complete a brief questionnaire where socio-demographic and brief health information was gathered. Informed written and oral consent was obtained from all participants and the study was approved by the regional ethical committee in Linköping, Sweden (Dnr: 2016/30–31).

### Physical activity assessment

Two different accelerometer models were used from the same manufacturer (Actigraph models GT1M and GT3X; ActiGraph. LLC Pensacola. FL). These have been shown to provide comparable outputs of physical activity recorded using the accelerometer’s vertical axis [23]. Therefore, only data recorded on the vertical axes of the accelerometers was used in this study.

The accelerometer was set to collect data using 5-s epochs. After data collection, the data was treated according to a commonly used procedure for non-wear time detection: i.e. for a day to be considered as valid, the wear time had to exceed > 600 min * day^− 1^, once periods of > 20 min of consecutive epochs with 0 counts had been removed [24]. Only those with at least 21 days of valid monitoring were included in the subsequent analysis. To calculate the duration of physical activity at different intensities the following cut-points were used: < 100 counts per minute (cpm) for sedentary behaviour [4], 100–1951 cpm for light physical activity, 1952–5723 cpm for moderate physical activity, 5724 and higher for vigorous physical activity [25]. “At least moderate intensity physical activity” (MVPA) was calculated as the sum of all epochs with 1952 cpm or more (i.e. moderate plus vigorous). Counts were calculated as the sum of all counts per day. The calculations was made using a custom python-script and then exported to R for further analysis.

### Data analysis

Four different data sets were created to illustrate different approaches to data collection. The first consisted of the full set of data, that is, all valid days for all subjects with 21 or more valid days of measurement. For the second dataset, a random within-subject sample of 7 days was drawn so that each subject got seven random days drawn from their own data. The third dataset consisted of the first 7 days of observations for each included subject. For the fourth dataset, 3 days were selected at random from the dataset consisting of the seven first days of measurement. The following calculations were made on each dataset separately.

Firstly, to estimate the SEM, the within- and between subject variances was estimated from a set of intra class correlations (ICC). The ICCs were calculated using the ICCest function from the R package ICC [26]. The ICCs were also used in order to make comparisons with previous studies predominantly conducted on level three (see supplementary files). The ICCest function is suited for unbalanced data with different number of observations within each subject and also provides the within- and between-subject variations needed for the calculation of SEM.

Secondly, the within- and between-subject variations estimated from the four different datasets and for the different intensity levels was entered in eq. 1 and kept fixed for different combinations of *n* and *m* [27].

1 $$ SEM=\sqrt{\frac{\sigma^2}{n}+\frac{\delta^2}{mn}} $$

In which *σ*^2^ is the between-subject variation and *δ*^2^ is the within-subject variation *m* is the number of measured days and *n* is the number of subjects.

The physical activity of the population was described using mean and standard deviation. In addition, the relative standard error was calculated as the SEM from eq. 1 divided by the mean value and expressed in percent.

## Results

Out of the 61 subjects who initially volunteered, 50 subjects (67% women, with a mean (SD) age of 41 [19] years) met the inclusion criteria of having at least 21 days of valid measurement. Table 1 describes the physical activity levels for the four different datasets. None of the observed differences between the datasets in the mean level of physical activity at the different intensities was significantly different from each other (ANOVA, *p* > 0.05).

**Table 1:** Descriptive data for the different datasets used in the study

	Mean		SD	Relative SEM
*All days*				
Sedentary	613.8	±	50.8	1.17%
Light PA	131.6	±	33.3	3.58%
Moderate PA	53.2	±	20.8	5.52%
Vigorous PA	6.6	±	6.3	13.56%
MVPA	59.8	±	23.2	5.48%
Counts	320,388	±	114,944	5.07%
*Three days*				
Sedentary	611.4	±	71.3	1.65%
Light PA	129.2	±	36.0	3.95%
Moderate PA	53.8	±	23.0	6.05%
Vigorous PA	5.8	±	7.5	18.19%
MVPA	59.6	±	27.5	6.52%
Counts	317,131	±	146,366	6.53%
*First seven days*				
Sedentary	601.2	±	58.0	1.39%
Light PA	132.8	±	38.2	4.06%
Moderate PA	53.5	±	22.8	6.02%
Vigorous PA	6.6	±	6.8	14.68%
MVPA	60.1	±	25.1	5.90%
Counts	321,375	±	117,984	5.57%
*Seven random days*				
Sedentary	608.7	±	59.9	1.39%
Light PA	132.5	±	32.9	3.51%
Moderate PA	52.9	±	22.7	6.07%
Vigorous PA	6.8	±	7.0	14.36%
MVPA	59.7	±	25.4	6.01%
Counts	321,807	±	126,700	5.57%

**Fig. 2:**
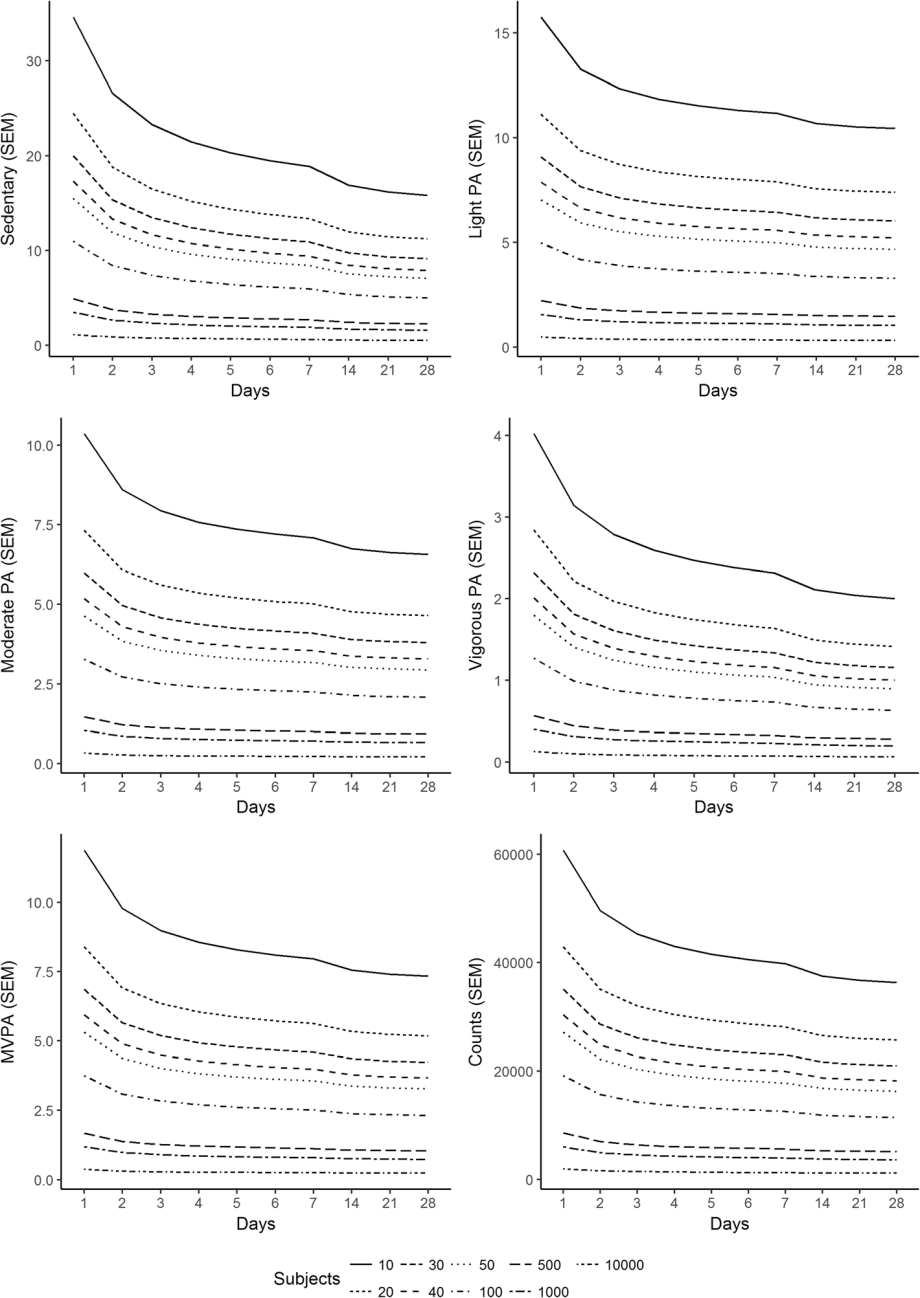
The effect of different combinations of repeated observations (days) or number of included subjects in the sample on SEM

Given the small differences in SEM between the different datasets, only the calculations for the dataset with all days is displayed but the pattern for the other datasets is identical (see supplementary files for the other results). In Fig. 2, the outcome of the calculations for the different combinations of days and subjects is shown. It shows that, independently of which intensity level of physical activity is of interest, increasing the number of subjects rather than the number of repeated observations within each subject has the greatest impact on the SEM. For example, for MVPA, collecting 1 day of data from 100 subjects (i.e. 100 observed days) produces an SEM that is similar to the SEM for seven repeated observations in 50 subjects (i.e. 350 observed days). And 2 days from 100 subjects (200 days) will produce an SEM that is smaller than 28 days from 50 subjects (1400 days).

The outcome of the calculations for level 3 studies are shown in the supplementary file.

## Discussion

The aim of this study was to investigate how different combinations of sample size and repeated observations within individuals influences SEM, and thus the ability to estimate the uncertainty of the population estimate of physical activity by confidence intervals or by detecting group differences with, for example, a t-test. This was done with the help of four different subsets mimicking different accelerometer-based measurement protocols: using all (21–28) valid days, seven random days of measurement, the first 7 days of measurement, and three random days of measurement from the first 7 days. The result shows that, to get as low an SEM as possible it’s more efficient in terms of total number of observed days to maximize the number of subjects and to keep the number of repeated observations within each subject close to one. This is the same conclusion as Lee, P.H. (2018) reached when they investigated how, given a fixed amount of accelerometers, varying the number of days and subjects influenced on the number days needed within the ICC framework, i.e. level 3 related questions [28]. It appears as if there has been a long-lasting misconception that there is a “one size fits all” protocol when it comes to physical activity measurement using accelerometers. However, as this study shows, there is no one protocol for accelerometer-based physical activity that is suitable for all possible research questions. The current standard protocol of 7 days of repeated measurement within each individual can most likely be traced back to one review, which is currently cited over one thousand times [4]. The authors suggested that to measure physical activity in adults and children, 3–5 and 4–9 days, respectively of monitoring was appropriate. They also stated that:

*For investigators, the goal is to monitor activity for a sufficient number of days so that the resulting daily average reflects an individual's usual or habitual level of physical activity* [4].

This statement holds true only if the research question is at level 4, identifying the habitual level of physical activity in an individual. This is not as important for studies at level 1–2 since the between subject variation will cancel each other out, e.g. some will be more active compared to their average and some will be less active, and the group level estimate will be valid if the sample size is sufficiently large [29]. In addition, the studies included in that review were in fact all attempting to determine the number of repeated days of measurement that was needed to rank individuals according to their level of physical activity, i.e. level 3. This illustrates the misconception regarding what it means to assess habitual physical activity of an individual. In other words, there is a difference between what is needed to describe the habitual physical activity of one person (*n* = 1) and to be able to rank this individual correctly according to their level of physical activity in a group of individuals (*n* > 1). The latter situation (level 3) is the one that most of the previous research has dealt with and the difference between the two can be illustrated as follows. To answer questions in level three situations one first calculates an ICC (or other appropriate measure such as in the case for G-theory). As a second step, one takes that and enters it into the Spearman-Brown prophecy formula to generalize into the number of measurements (days) that is needed to, with a desired reliability, be able to correctly rank individuals according to their level of physical activity. The Spearman-Brown prophecy formula is used to estimate the number of repeated observations needed to rank individuals to a desired level of reliability according to:

2 $$ D=\frac{ICC_d\ast \left(1-{ICC}_o\right)}{ICC_o\ast \left(1-{ICC}_d\right)} $$

In which ICCd is the desired reliability (e.g. 0.8) and ICCs is the observed reliability in the group. The outcome of that calculation based on the current sample can be found in the supplementary file.

This procedure will not generalize to estimate the number of observations needed to identify the habitual physical activity of one single individual.

This becomes obvious when looking at the formulas. Consider the following situation. The ICC is calculated (depending on which ICC in the larger family of ICCs) as for example $\mathrm{ICC}=\frac{\upsigma_{\mathrm{b}}^2}{\upsigma_{\mathrm{b}}^2+{\upsigma}_{\mathrm{w}}^2}$ where ${\upsigma}_{\mathrm{b}}^2$ is the between-subject variation and ${\upsigma}_{\mathrm{w}}^2$ is the within-subject variation. If ${\upsigma}_{\mathrm{b}}^2$ = 100 and ${\upsigma}_{\mathrm{w}}^2$ = 25 then the ICC = 0.8. However, if ${\upsigma}_{\mathrm{b}}^2$ = 10 and ${\upsigma}_{\mathrm{w}}^2$ = 2.5 then the ICC is still 0.8 even if the within-subject variation differs by a factor of 10.

In both situations the ICC becomes identical, and by extension so will the outcome from Spearman-Brown prophecy formula. However, the within and between subject variations differ by a factor of 10 between the situations, which will certainly change the ability to identify the habitual physical activity of an individual. The habitual level of physical activity can be defined, slightly modified from Lui et al’s version for diet, as: “the hypothetical average around which that individual’s physical activity varies” [30]. To estimate the habitual level of an individual it is therefore necessary to estimate the within subject coefficient of variation. That is, how much does an individual fluctuate around their true, but unmeasured, mean level of physical activity. That value is then entered in eq. 3

3 $$ D={\left(\frac{Z\alpha \ast CVw\ }{D_0}\right)}^2 $$

In which D is the number of days needed to monitor. Zα is the normal deviate for which the percentage of time the measured value should fall within a specified limit (i.e 1.96 = 95% confidence). CVw is the within-subject coefficient of variation, and D_0_ is how close to the “true” habitual level the observed value should fall (e.g. 20%). The outcome from such an analysis is interpreted as the number of repeated observations needed so that the observed value is within ±20% of the true habitual level 95% of the time.

We have previously published work that estimated how many days are needed to estimate the usual physical activity of an individual (level 4). We showed that, for most intensity levels, considerably more days are needed than for research questions on level 3 [22].

Thus, if the results of the present study are combined with previous studies in this field [5–19, 22], a more nuanced picture than a “one size fits all” emerges when it comes to protocols for accelerometer-based physical activity assessment. Depending on which research question is to be answered there are several decisions that the researcher needs to make, including about the number of subjects to be included in the study and the number of repeated observations within each of the included subjects. However, the optimal accelerometry-based assessment protocol will also have to factor in other considerations, such as the subject burden of wearing accelerometers, the costs of including either more subjects or more days per subject and so on. The researcher must determine the best protocol given all of these different circumstances. The present study together with the other studies in the field should make it easier for a researcher to make informed decisions regarding these questions.

The study population could be viewed as a potential limitation to the results as they were not selected at random and were more active compared to the general Swedish population [31]. However, the outcome of the study is independent of the studied population or their level of physical activity. The same conclusion, that it is more efficient to reduce the SEM by including more subjects than increasing repeated observation within each subject, would have been reached if we would have simulated the data from scratch. However, the level of the SEM will change in different populations and it is therefore important to make power calculations with high quality information on the relevant population at hand before designing a study.

Another issue that goes beyond the scope of this study is the influence of other sources of variance that effects on the outcome such as day of the week effect [7], seasonal variations [32, 33], even if this variation may be trivial on group level [34], to physical activity or other sources related to for example gender and age distribution in the population. To get population estimates of physical activity levels within for example a national monitoring system it’s important to consider these factors and not only days versus subjects when choosing an appropriate protocol and when sampling the study participants from the target population, and the best way to do so may to use a simple single sample selection procedure and not to force any combination [35].

## Conclusion

This study shows that it is more efficient, in terms of keeping the number of observed days to a minimum, to reduce the SEM by maximising the number of subjects in a study rather than increasing the number of repeated measurements within each subject. Thus, for a study in which the aim is to estimate a population prevalence of physical activity or a study designed to compare groups it is more efficient to have large sample sizes with few repeated observations within each subject. Even though the importance of reducing the SEM to increase the ability to detect differences between groups is well-known it is seldom considered when developing appropriate protocols for accelerometer based research. Therefore, the results presented herein serves to highlight this fact and have the potential to stimulate debate and challenge current best practice recommendations of accelerometer based physical activity research.

## Supplementary information

**Additional file 1.** Supplementary material 1. Outcome from ICC and level 3 calculations. Supplementary material 2. the outcome from the SEM calculations for random seven days, the first week of measurement and three days from the first week of measurement.

## Data Availability

The datasets generated during and/or analysed during the current study are available in the Open Science Framework repository https://osf.io/4ttfq/

## Abbreviations

ANOVA: ANalysis Of VAriance.
CI: Confidence Interval.
CPM: Counts Per Minute.
ICC: Intra Class Correlation.
SD: Standard Deviation.
SEM: Standard error of the mean.
