# Supplementary material for: No one accelerometer-based physical activity data collection protocol can fit all research questions
Source: BMC Med Res Methodol. 2020 Jun 3;20:141. doi: 10.1186/s12874-020-01026-7 (PMC7271555; doi:10.1186/s12874-020-01026-7)
Supplement: Supplementary file 1 — Additional file 1. Supplementary material 1. Outcome from ICC and level 3 calculations. Supplementary material 2. the outcome from the SEM calculations for random seven days, the first week of measurement and three days from the first week of measurement. [file 12874_2020_1026_MOESM1_ESM.docx]

SUPPLEMENTARY MATERIAL

**Supplementary material 1 - Outcome from ICC and level 3 calculations**

For the sake of comparisons with previous studies, predominantly conducted on level 3, a set of intra class correlations (ICC) was calculated. The ICCs were calculated using the ICCest function from the R package ICC (Wolak, Fairbairn, & Paulsen, 2012). The ICCest function is suited for unbalanced data with different numbers of observations within each subject and also provides the within- and between-subject variations needed for the calculation of SEM. The ICCs were then entered in the Spearman-Brown prophecy formula (Mattocks et al., 2008). The Spearman-Brown prophecy formula is used to estimate the number of repeated observations needed to rank individuals to a desired level of reliability according to:

$$D= \frac{{ICC}_{d}*{(1-ICC}_{o})}{{ICC}_{o}*{(1-ICC}_{d})}$$

ICC_d_ is the desired reliability which was in this study set to 0.7, 0.8 and 0.9. ICC_o_ is the observed ICC which was set to 0.2-0.6 according to the range of ICCs estimated from the ICCest-function.

The observed ICC for each intensity of physical activity as well as for total number of counts is shown in Figure 1.


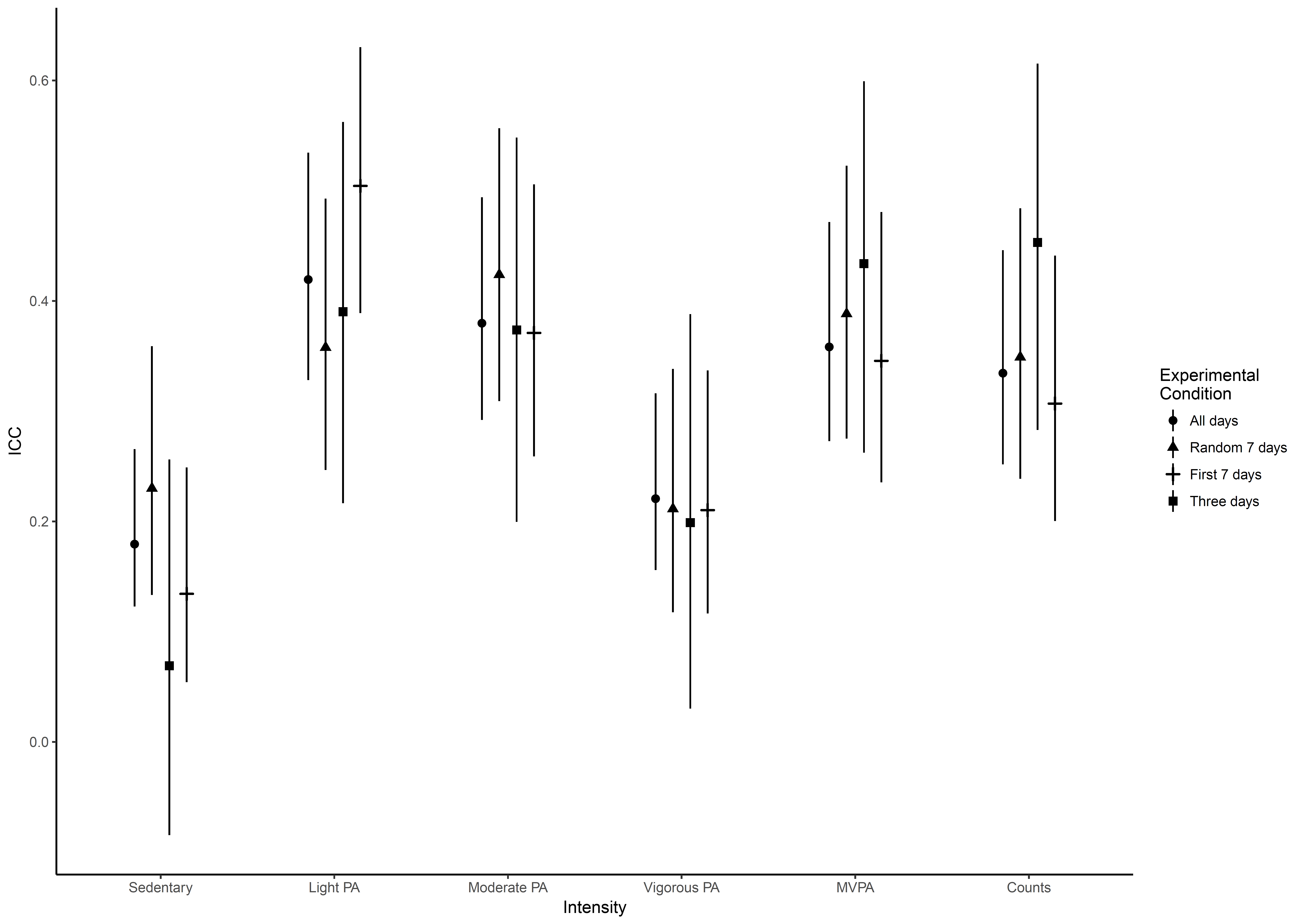


**Figure 2** The observed ICC and its associated 95% confidence intervals for different experimental settings

The observed ICC from Figure 1 should then be compared with the calculations shown in Figure 2 to get an idea of the number of repeated observations needed to be able to rank the individuals between each other with a desired level of reliability. For example, the observed ICC for MVPA seems to cluster around 0.35-0.40. This corresponds to approximately 5 days of repeated measurements are needed to, with a reliability of 0.7, rank the individuals according to their level of MVPA.


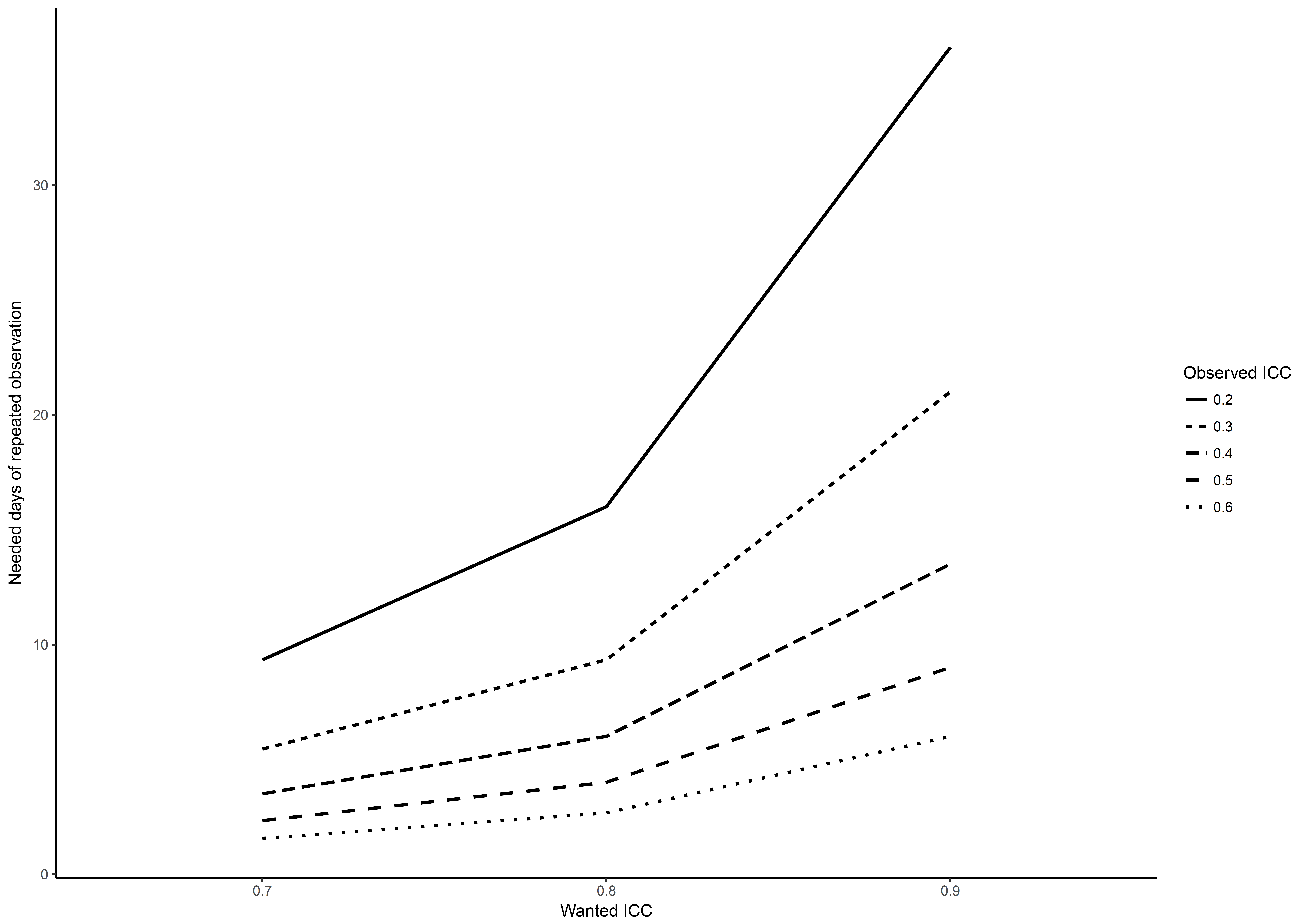


Figure 2: The number of repeated days of observations needed to rank individuals according to their level of physical activity to a desired level of reliability given the observed reliability.

References

Mattocks, C., Ness, A., Leary, S., Tilling, K., Blair, S. N., Shield, J., … Riddoch, C. (2008). Use of accelerometers in a large field-based study of children: protocols, design issues, and effects on precision. *J Phys Act Health*, *5 Suppl 1*, S98-111.

Wolak, M. E., Fairbairn, D. J., & Paulsen, Y. R. (2012). Guidelines for estimating repeatability. *Methods in Ecology and Evolution*, *3*(1), 129–137. https://doi.org/10.1111/j.2041-210X.2011.00125.x

**Supplementary material 2 - the outcome from the SEM calculations for random seven days, the first week of measurement and three days from the first week of measurement.**


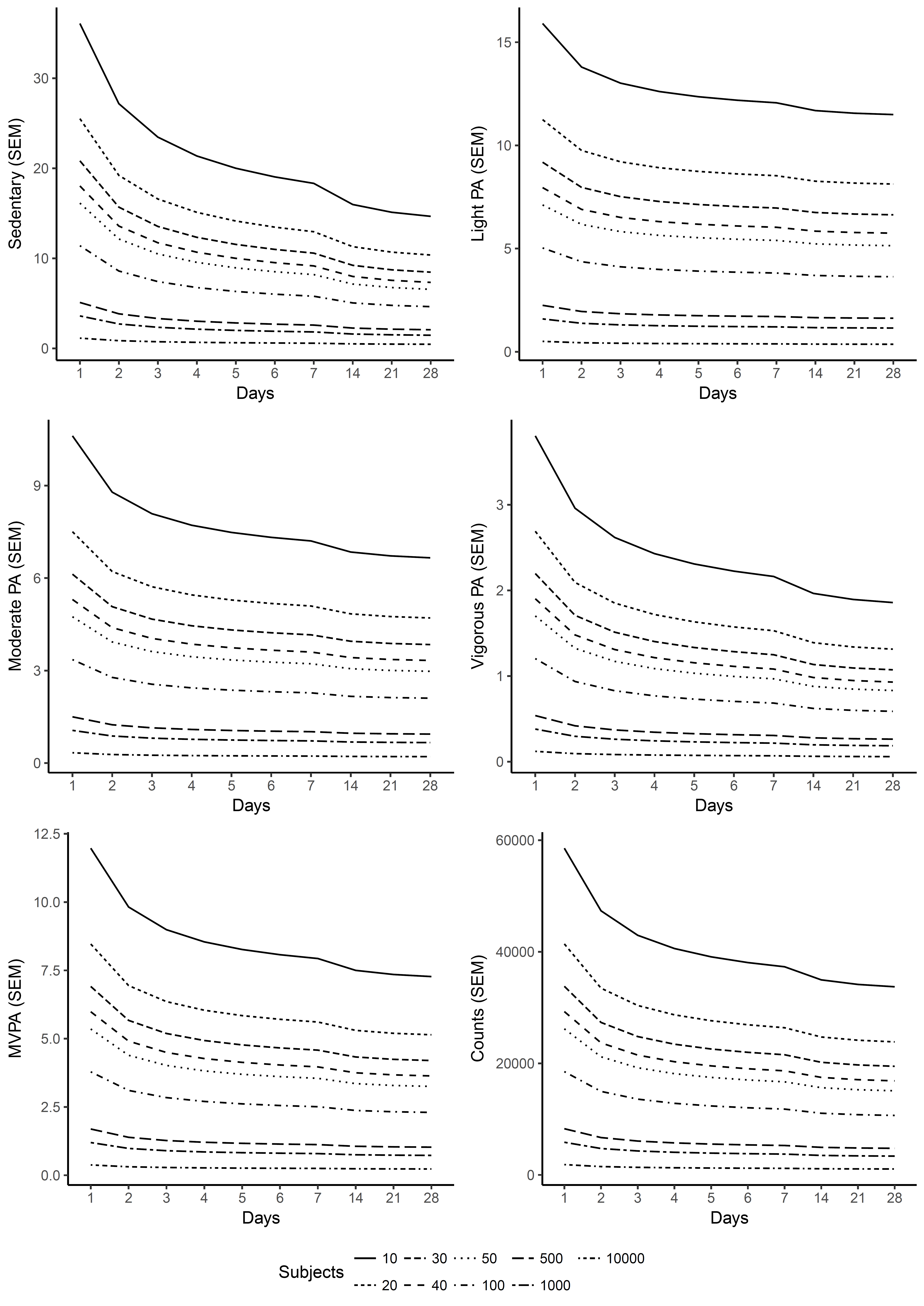


Figure 1 The effect of different combinations of repeated observations (days) or number of included subjects in the sample on SEM. The calculations are made for the dataset containing a within subject random sample of seven days of measurement.


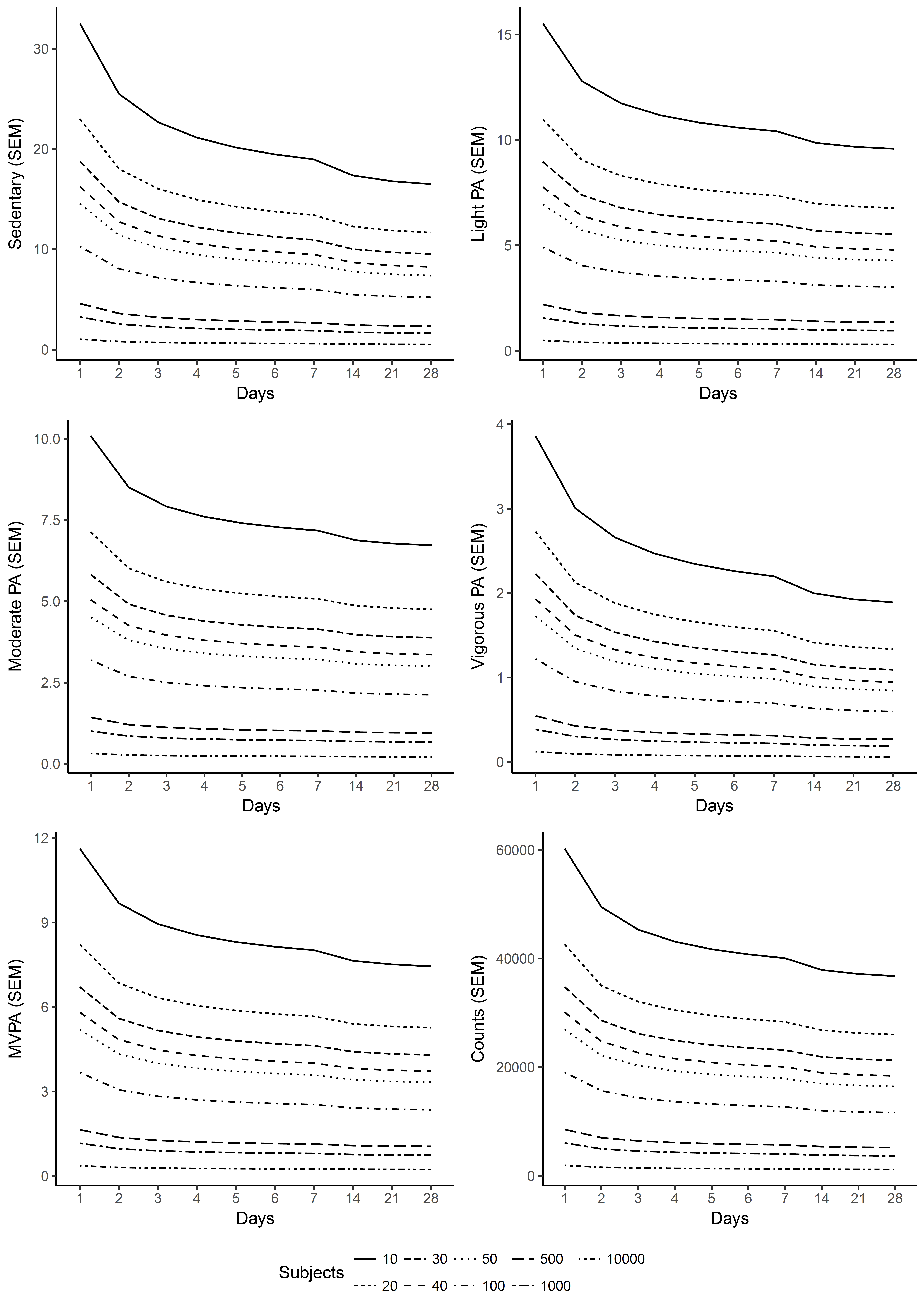


Figure 2 The effect of different combinations of repeated observations (days) or number of included subjects in the sample on SEM. The calculations are made for the dataset containing the first seven days of measurement.


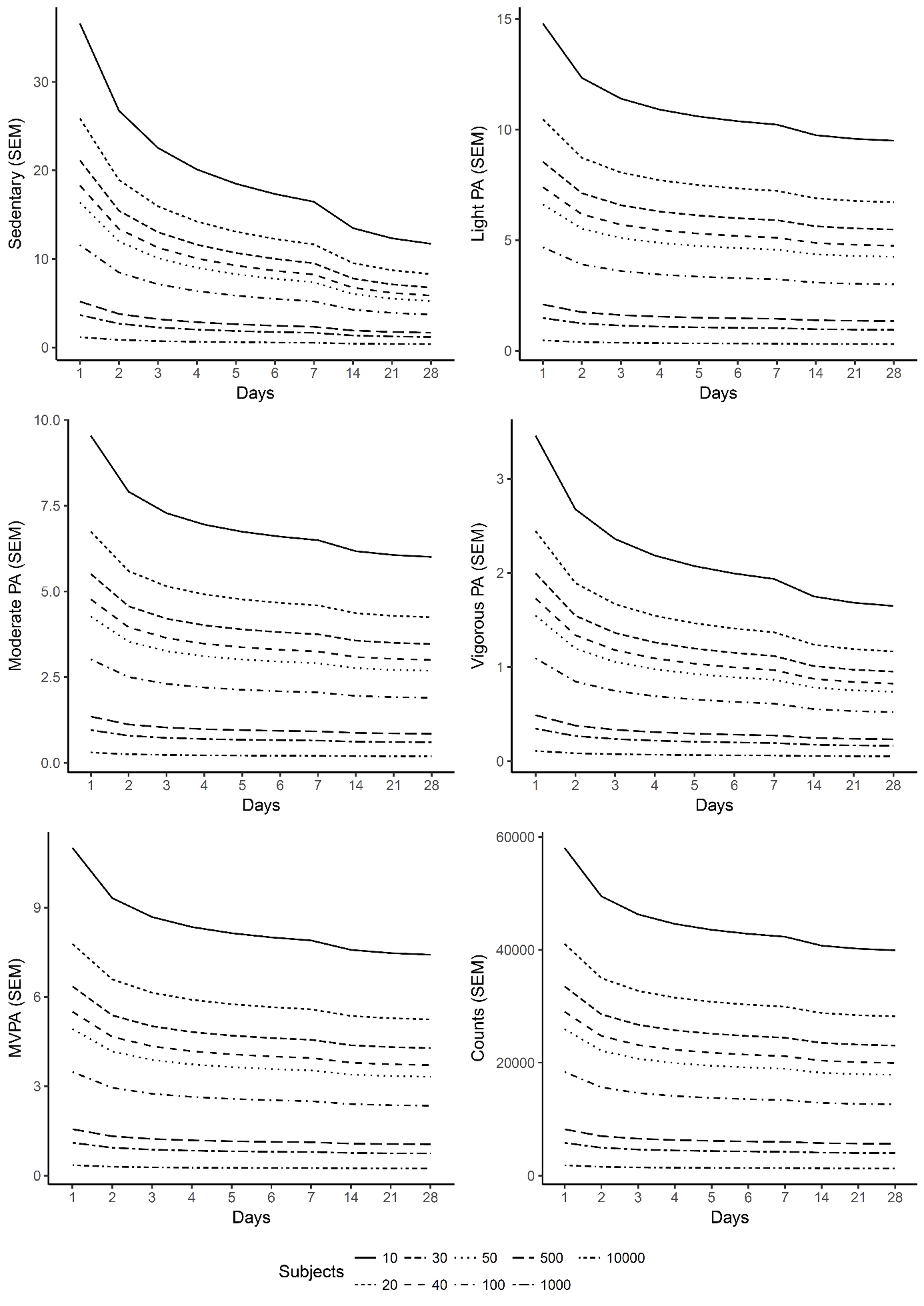
Figure 3 The effect of different combinations of repeated observations (days) or number of included subjects in the sample on SEM. The calculations are made for the dataset containing three days of measurement randomly selected from the first seven days.
